# Perioperative Quality Initiative (POQI) consensus statement on fundamental concepts in perioperative fluid management: fluid responsiveness and venous capacitance

**DOI:** 10.1186/s13741-020-00142-8

**Published:** 2020-04-21

**Authors:** Greg S. Martin, David A. Kaufman, Paul E. Marik, Nathan I. Shapiro, Denny Z. H. Levett, John Whittle, David B. MacLeod, Desiree Chappell, Jonathan Lacey, Tom Woodcock, Kay Mitchell, Manu L. N. G. Malbrain, Tom M. Woodcock, Daniel Martin, Chris H. E. Imray, Michael W. Manning, Henry Howe, Michael P. W. Grocott, Monty G. Mythen, Tong J. Gan, Timothy E. Miller

**Affiliations:** 1 Division of Pulmonary, Allergy, Critical Care and Sleep Medicine, Emory Critical Care Center, Emory University School of Medicine, Grady Health System, Atlanta, GA USA; 2 Division of Pulmonary, Critical Care, and Sleep Medicine, NYU School of Medicine, New York, NY USA; 3 Division of Pulmonary and Critical Care Medicine, Eastern Virginia Medical School, Norfolk, VA USA; 4 Department of Emergency Medicine, Beth Israel Deaconess Medical Center, Boston, MA USA; 5 Critical Care Research Group, NIHR Biomedical Research Centre, University Hospital Southampton NHS Foundation Trust/University of Southampton, Southampton, UK; 6 Department of Anesthesiology, Division of General, Vascular and Transplant Anesthesia, Duke University School of Medicine, Duke University Medical Center, Durham, NC USA; 7 TopMedTalk, London, UK; 8 Institute of Sport Exercise & Health, University College London, London, UK; 9 University Hospitals Southampton, Southampton, UK; 10 Respiratory Biomedical Research Unit, University of Southampton, Southampton, England; 11 Department of Intensive Care, University Hospital Brussels, Jette, Belgium and Facultyof Medicine and Pharmacy, Vrije Universiteit Brussels, Brussels, Belgium; 12 Elsevier R&D Solutions, 1600 JFK Blvd, Philadelphia, PA 19103 USA; 13 Intensive Care Unit and Division of Surgery and Interventional Science, Royal Free Hospital, London, UK; 14 Vascular and Renal Tranplant Surgeon, National Institute of Health Research Clinical Research Facility, Coventry, UK; 15 UCL/UCLH National Institute of Health Research Biomedical Research Centre, London, UK; 16 Department of Anesthesiology, Stony Brook University, Stony Brook, NY USA; 17 Department of Anesthesiology and Critical Care, Stony Brook University, Stony Brook, New York, USA; 18 Private address: Louisville, Kentucky, USA

**Keywords:** Perioperative fluid management, Physiology, Fluid responsiveness, Venous capacitance, Goal-directed fluid therapy

## Abstract

**Background:**

Optimal fluid therapy in the perioperative and critical care settings depends on understanding the underlying cardiovascular physiology and individualizing assessment of the dynamic patient state.

**Methods:**

The Perioperative Quality Initiative (POQI-5) consensus conference brought together an international team of multidisciplinary experts to survey and evaluate the literature on the physiology of volume responsiveness and perioperative fluid management. The group used a modified Delphi method to develop consensus statements applicable to the physiologically based management of intravenous fluid therapy in the perioperative setting.

**Discussion:**

We discussed the clinical and physiological evidence underlying fluid responsiveness and venous capacitance as relevant factors in fluid management and developed consensus statements with clinical implications for a broad group of clinicians involved in intravenous fluid therapy. Two key concepts emerged as follows: (1) The ultimate goal of fluid therapy and hemodynamic management is to support the conditions that enable normal cellular metabolic function in order to produce optimal patient outcomes, and (2) optimal fluid and hemodynamic management is dependent on an understanding of the relationship between pressure, volume, and flow in a dynamic system which is distensible with variable elastance and capacitance properties.

## Consensus statements

### Physiological principles of fluid resuscitation

1. The ultimate goal of fluid and hemodynamic management is to support normal cellular metabolic function.
2. Achieving normal cellular metabolic function requires maintenance or restoration of effective coordinated function of the macrocirculation and the microcirculation, as well as intact cellular metabolism.
3. Practically speaking, most clinical management is currently targeted at macrocirculatory variables and surrogates of cellular metabolism (e.g., lactate, base excess).
4. The therapeutic rationale of intravenous fluid administration is to optimize macrocirculatory function in order to improve or optimize microcirculatory and cellular function.
5. There is a minimal intravascular volume required to maintain cardiac output and stroke volume and normal tissue perfusion. Below this volume, cardiac output and blood pressure may be maintained at the expense of microcirculatory blood flow and cellular function.
6. The majority of intravascular volume is in the venous circulation; therefore, the venous capacitance is a critical determinant of effective macrocirculatory function.

### Physiology of fluid responsiveness

1. There is no readily available method of measuring intravascular volume and it is uncertain if knowing this static value would have clinical utility.
2. Optimal intravascular volume can only be characterized through dynamic evaluation.
3. Administration of a fluid bolus as part of a fluid challenge is a means of increasing intravascular volume to evaluate the effect on stroke volume.
4. Fluid responsiveness is defined as a state of recruitable stroke volume in response to intravascular fluid administration.

### Venous capacitance

1. The venous circulation is comprised of stressed and unstressed volumes.
2. Stressed volume is the (theoretically measurable) volume of blood that exerts distending pressure against the venous wall. In contrast, unstressed volume is the volume of blood up to the point of filling the veins but without exerting any pressure on the vessel walls.
3. Stressed volume determines the mean systemic filling pressure (MSFP, the pressure of venous return when cardiac activity is absent), related to the elastic recoil of the venous system. The difference between the MSFP and right atrial pressure is the major factor determining venous return to the heart. The MSFP provides driving pressure against right atrial pressure which creates a gradient promoting forward flow.

### Practical implications

1. Full characterization of fluid responsiveness requires consideration of the type, amount and timing of fluid as well as the expected change in stroke volume.
2. The best method of measuring fluid responsiveness is a continuous or rapidly repeatable measure of stroke volume.
3. A common approach to test fluid responsiveness is the administration of 250-500 mL bulos in < 15 min with a positive response defined by a 10-15% increase in stroke volume.
4. The passive leg-raise maneuver replicates a transient fluid bolus and predicts fluid responsiveness without administration of intravenous (IV) fluids (positive response definted as > 10% increase in stroke volume), thereby mitigating the risks of excess IV fluid administration. This maneuver has limited utility in the intraoperative setting.
5. Alternative methods for predicting fluid responsiveness include stroke volume variation (SVV), pulse pressure variation (PPV), systolic pressure variation (SPV), and (in certain mechanically ventilated patients) end-expiratory occlusion test and respiratory systolic variation test. All have limitations (Table 1).
6. Sonographic evaluation of IVC size, distensibility, or collapsibility has limited and unproven utility at the present time.
7. The actions of vasoactive drugs are typically considered in relation to the arterial circulation but many have significant effects on the venous circulation.
8. Venoconstrictors (e.g., alpha agonists) increase venous tone and thereby reduce venous capacitance, thus increasing the stressed volume at the expense of the unstressed volume. In a hypovolemic patient, this reduction in unstressed volume may in turn reduce microcirculatory blood flow and thereby compromise cellular metabolism, due to reduced perfusion despite maintenance of normal blood pressure.
9. Venodilators (e.g., nitroglycerin) reduce venous tone and thereby increase venous capacitance and decrease the stressed volume. This typically decreases venous return and left ventricular end-diastolic volume.
10. Intra-abdominal hypertension (e.g., pneumoperitoneum) may reduce venous return or venous capacitance.

**Table 1:** Summary of methods predicting fluid responsiveness

Method	Threshold (%)	Main limitations
Pulse pressure/stroke volume variations (PPV/SVV) (Michard et al., 2000)	12	Cannot be used in case of spontaneous breathing, low tidal volume/lung compliance. Need regular cardiac rhythm
Inferior vena cava diameter variations (Vignon et al., 2017)	12	Cannot be used in case of spontaneous breathing, low tidal volume/lung compliance. Need regular cardiac rhythm
Superior vena cava diameter variations (Vignon et al., 2017)	36	Requires performing transesophageal Doppler. Cannot be used in case of spontaneous breathing, low tidal volume/lung compliance. Need regular cardiac rhythm
Passive leg raising (Monnet et al., 2006)	10	Requires a direct measurement of cardiac output. May be inaccurate in intra-abdominal hypertension
End-expiratory occlusion test (Monnet et al., 2009)	5	Cannot be used in non-intubated patients. Cannot be used in patients who interrupt a 15-second respiratory hold
“Mini” fluid challenge (Muller et al., 2011)	6	Requires a precise technique for measuring cardiac output
“Conventional” fluid challenge (500 mL) (Vincent & Weil, 2006)	15	Requires a direct measurement of cardiac output. Can induce fluid overload if repeated

## Background

More than 230 million major surgical procedures are undertaken worldwide each year (Weiser et al., 2008). Data from the USA and Europe suggests that approximately 18% of patients undergoing surgery will develop a major postoperative complication and 3 to 5% will die before hospital discharge (Weiser et al., 2008; Khuri et al., 2005; Ghaferi et al., 2009; Pearse et al., 2012). Those patients who develop a postoperative complication and survive to hospital discharge have diminished functional independence and reduced long-term survival up to 8 years after major surgery (Khuri et al., 2005). Interventions that reduce the risks of postoperative death and complications, particularly in high risk patients have become a priority in perioperative medicine (Jacobs, 2009). Perioperative goal-directed therapy (GDT), based on the titration of fluids and vasoactive drugs to achieve physiological, flow-related end points, is a promising approach to reduce postoperative complications and deaths (Bednarczyk et al., 2017; Pearse et al., 2014; Hamilton et al., 2011).

Optimal fluid therapy in the perioperative and critical care settings depends on understanding the underlying cardiovascular physiology and individualizing assessment of the dynamic patient (Malbrain et al., 2018). Historical approaches to fluid administration based on clinical examination (e.g., “volume status”) or static measures of cardiovascular function (e.g., central venous pressure or pulmonary artery occlusion pressure) do not adequately determine fluid needs or to predict response to fluid administration (Van der Mullen et al., 2018). Dynamic indices, such as SVV, utilize the concepts of the Frank-Starling relationship that implicitly incorporate venous capacitance and mean systemic filling pressure to predict fluid responsiveness with good accuracy. However, in the context of optimal fluid administration, knowledge gaps remain for several key physiologic concepts and clinical scenarios. This manuscript from the 5th Perioperative Quality Initiative (POQI) group addresses fundamental concepts in fluid responsiveness and venous capacitance in order to provide an educational update and clinical guidance related to perioperative fluid management.

## Methods

POQI is an international, multidisciplinary non-profit organization that organizes consensus conferences on clinical topics related to perioperative medicine (Miller et al., 2016). Each conference assembles a collaborative group of diverse international experts from multiple healthcare disciplines to develop consensus-based recommendations in perioperative medicine.

Applying a modified Delphi method, designed to use the collective expertise of a diverse group of experts to answer clinically important questions, we achieved consensus on several topics related to fluid responsiveness and fluid management.

### Expert group

An international group of authorities, with specific content area expertise (based on the conduct of research and education in this area), was invited to participate. In total, 21 experts from around North America and Europe met in Durham, NC, on June 16-17, 2018, to iteratively discuss the clinical and physiological evidence of fluid responsiveness and venous capacitance as relevant factors in fluid management, in order to develop consensus statements with practical recommendations for a broad group of clinicians involved in intravenous fluid therapy.

### Process

Based on literature searches performed by POQI group members, a list of relevant questions was collectively formulated and circulated electronically prior to the meeting. In the first plenary session, these questions were presented to receive feedback and assistance in refining the questions. There were then at least two Delphi rounds to develop the statements before final agreement. This manuscript is based on these multiple rounds of feedback from all the experts present at the POQI meeting.

## Results/discussion

Key physiologic and clinical terminology are shown in Tables 2 and 3. Based on the literature identified by the participants and discussions held both prior to the conference and during the iterative consensus-building process, the following key concepts and core questions were considered most relevant to perioperative fluid management with respect to fluid responsiveness and venous capacitance:

**Table 2:** Physiologic terminology

Term	Definition
Arterial elastance	The ratio of left ventricular end-systolic pressure and stroke volume
Intravascular volume	The blood volume within the vascular system (arteries, capillaries, veins)
Mean systemic filling pressure	The pressure of venous return when cardiac activity is absent
Preload	Volume defined by the distending pressure it generates. In the heart, preload is LV wall stress at end of diastole (= EDV)
Stressed volume	The (theoretically measurable) volume of blood that exerts distending pressure against the vascular wall
Total body water	The amount of sodium-free water in the whole body, commonly divided into the extracellular fluid space and the intracellular fluid space
Unstressed volume	The volume of blood just to the point of filling the blood vessels but without exerting any pressure on the vessel walls
Vascular capacitance	The change in volume divided by the change in pressure (i.e., the inverse of elastance)

**Table 3:** Clinical terminology

Term	Definition
Fluid bolus	The rapid administration of intravenous fluid with therapeutic intent, most often to rapidly replace intravascular volume in patients who are presumed to be fluid responsive.
Fluid challenge	The rapid administration of intravenous fluid with diagnostic intent, most often to determine whether a patient with hemodynamic compromise will benefit from further fluid administration.
Fluid overload (overhydration)	Increased total body fluid volume (intravascular, interstitial, and intracellular). Fluid overload may be defined by at least 10% increase in total body fluid volume. Sometimes referred to as “overhydration” or “hyperhydration.” Fluid overload is the opposite of dehydration.
Fluid underload (dehydrataion)	Decreased total body fluid volume. The percentage of fluid loss is defined by dividing the cumulative fluid balance in liters by the patient’s baseline body weight and multiplying by 100%. Dehydration is defined by a minimum value of 5% fluid loss. Dehydration is considered mild (5-7.5%), moderate (7.5-10%), while loss of over 10% is considered severe. Sometimes referred to as “fluid underload.” Dehydration is the opposite of fluid overload.
Fluid responsiveness	An increase in stroke volume in response to an increase in intravascular volume. Also referred to as “volume responsiveness.”
Hypovolemia	Reduced intravascular volume and marked by increases in stroke volume when intravenous fluid is given (i.e., the state of being fluid responsive). Clinical “hypovolemia” may exist, for example, from loss of intravascular volume (e.g., hemorrhage) or from reductions in intravascular volume due to increases in venous capacitance. Sometimes referred to as “fluid underload.”
Hypervolemia	Hypervolemia is above normal or increased intravascular volume. Hypervolemia is the opposite of hypovolemia.
Passive leg raise	A diagnostic postural maneuver raising the lower extremities up to 45 degrees from the recumbent position, to transiently increase venous return from the lower extremities in order to measure the hemodynamic effect and thus determine if a patient is fluid responsive.

### Key concepts

I. *The ultimate goal of fluid therapy and hemodynamic management is to provide the conditions that enable normal homeostasis and cellular metabolic function in order to produce optimal patient outcomes.*
II. *Fluid and hemodynamic management is dependent on the relationship between pressure, volume, and flow in a dynamic system which is distensible with variable elastance and capacitance properties.*

### Core questions

#### What are the physiologic and clinical goals of therapeutic fluid (hemodynamic) resuscitation?

**Fig. 1:**
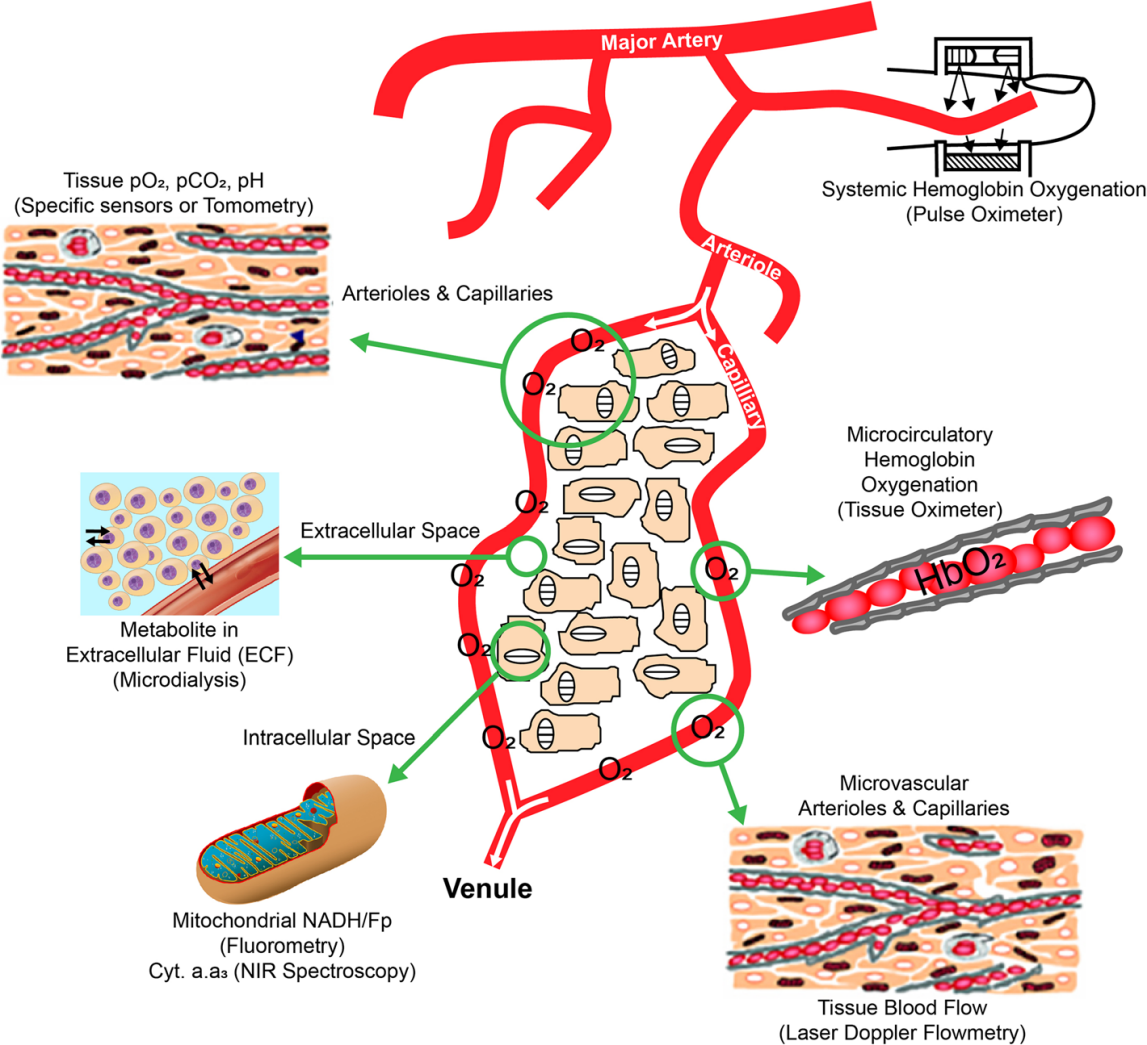
The macrocirculation, microcirculation, and the cellular level relevant for fluid therapy. Figure reused with the permission of the Perioperative Quality Initiative (POQI). For permission requests, contact info@poqi.org

The ultimate goal of fluid therapy and hemodynamic management is to provide the conditions that enable normal cellular metabolic function in order to produce optimal patient outcomes. The discrete goals of fluid therapy exist at several levels: at the level of the macrocirculation, the microcirculation, and at the cellular level (Fig. 1). It is a limitation of medical science that cellular metabolic function cannot be discretely and specifically measured in the clinical context, particularly locally for each of the wide variety of organ systems, and in continuous or repeatable series that permit clinical decision-making. Because of this limitation, for clinical care, we target intermediate variables of varying sensitivity, such as cardiac output (CO), stroke volume (SV), mean arterial pressure (MAP), central venous pressure (CVP), mixed venous saturation (SvO_2_) and central venous oxygen saturation (ScvO_2_), heart rate (HR), and urine output. While these parameters may indicate the presence of, or the risk for, cellular metabolic dysfunction, they are insensitive in this regard and do not differentiate cellular dysfunction due to macrocirculatory versus microcirculatory abnormalities.

Although restoration of the macrocirculation provides the basis for normal microcirculatory and cellular metabolic function, it does not guarantee them. Microcirculatory and cellular metabolic dysfunction may develop or persist despite establishing normal microcirculatory parameters, such as MAP and CO. However, because dysfunction of the macrocirculation (e.g., hypotension) often produces dysfunction at the microcirculatory and cellular levels, the first step in therapeutic fluid resuscitation is restoration of the macrocirculation. It is worth noting that restoration of the macrocirculation is not achieved by reaching the same target in every patient. Macrocirculatory parameters such as MAP and CO should be personalized both for the individual and for the patients’ current condition.

Recently, several investigators introduced the term “hemodynamic coherence” to describe the physiologic state in which improved macrocirculatory function results in improvements in the microcirculation (Ince, 2015; Morelli & Passariello, 2016). In contrast, the term “hemodynamic incoherence” describes a physiological state in which resuscitation to adequate macrocirculatory parameters does not result in an improved microcirculation. This state appears to happen frequently in patients with sepsis. Four subsets of hemodynamic incoherence are proposed. In the first, obstruction of some small blood vessels results in heterogeneous perfusion of the microcirculation. In the second, hemodilution results in perfusion of capillaries with blood that has a low oxygen carrying capacity. In the third, increased arterial resistance and increased venous pressures lead to capillary stasis due to low arterial-venous pressure gradients (see below). In the fourth, edema causes large distances between capillaries and target tissues across which oxygen and other energy substrates must diffuse to reach their targets. Notably, administration of IV fluid may lead to hemodilution, increased venous pressures, and edema formation, thus contributing to hemodynamic incoherence.

The first goal of therapeutic fluid and hemodynamic resuscitation is targeted at macrocirculatory parameters (usually MAP) because they are measurable and they represent the most apparent clinical markers of organ or tissue perfusion, particularly in combination with other clinical and laboratory assessments. When the MAP falls below an organ’s autoregulatory range, there is an almost linear decrease in organ blood flow (Ackland et al., 2019). The fall in blood flow is likely to occur at a higher MAP in patients with long-standing hypertension due to a shift in the autoregulatory range. Furthermore, different vascular beds will lose autoregulation at different MAPs. For example, the mammalian kidney loses autoregulation at a MAP of about 70 mmHg, the brain between 60-70 mm Hg, while the coronary circulation loses autoregulation at a MAP of about 50-55 mmHg (Drummond, 1997; Drummond, 2019; Paulson et al., 1990; Bellomo & Di Giantomasso, 2001; Meng, 2019). Therefore**,** the first hemodynamic goal is to achieve a MAP > 65-70 mmHg to preserve organ perfusion (Sessler et al., 2019).

The second and third goals of fluid and hemodynamic resuscitation are targeted at the microcirculation and the cellular levels. As noted above, dysfunction at these levels may develop or persist despite the appearance of normal macrocirculatory parameters, and devices to characterize the microcirculation and cellular function are not routinely available for clinical use. The microcirculation may be assessed directly using tools such as intravital microscopy or laser Doppler flowmetry, or indirectly using tissue oximetry and near infrared spectroscopy (Tafner et al., 2017). Clinical examination (e.g., examination of capillary refill time or assessing for skin mottling) may also give important clues about the adequacy of perfusion in the microcirculation (Hernandez et al., 2019). Efforts to realize the potential for microcirculatory monitoring for point-of-care diagnosis in real-time at the bedside are currently underway (Naumann et al., 2016).

In the clinical setting, the only reason to give any patient a fluid bolus is to increase the SV. In the absence of an increase in SV, giving a fluid challenge serves no useful purpose and is likely to be harmful. An increase in SV will only occur if two conditions are met: (1) that the fluid bolus increases the stressed blood volume causing an increase in MSFP, thereby increasing venous return, and (2) that both ventricles are functioning on the ascending limb of the Frank-Starling curve.

Organ blood flow is driven by the difference in the pressure between the arterial and venous circulation. For example, the MAP minus the CVP is the driving force for organ blood flow while the difference between post-arteriolar and venular pressure determines microcirculatory flow. In circumstances of increased venous pressure, such as high right atrial pressure, the backwards transmission of pressure may impede microcirculatory flow in the tissues and organs.

#### What is the role of venous capacitance in fluid and hemodynamic therapy?

**Fig. 2:**
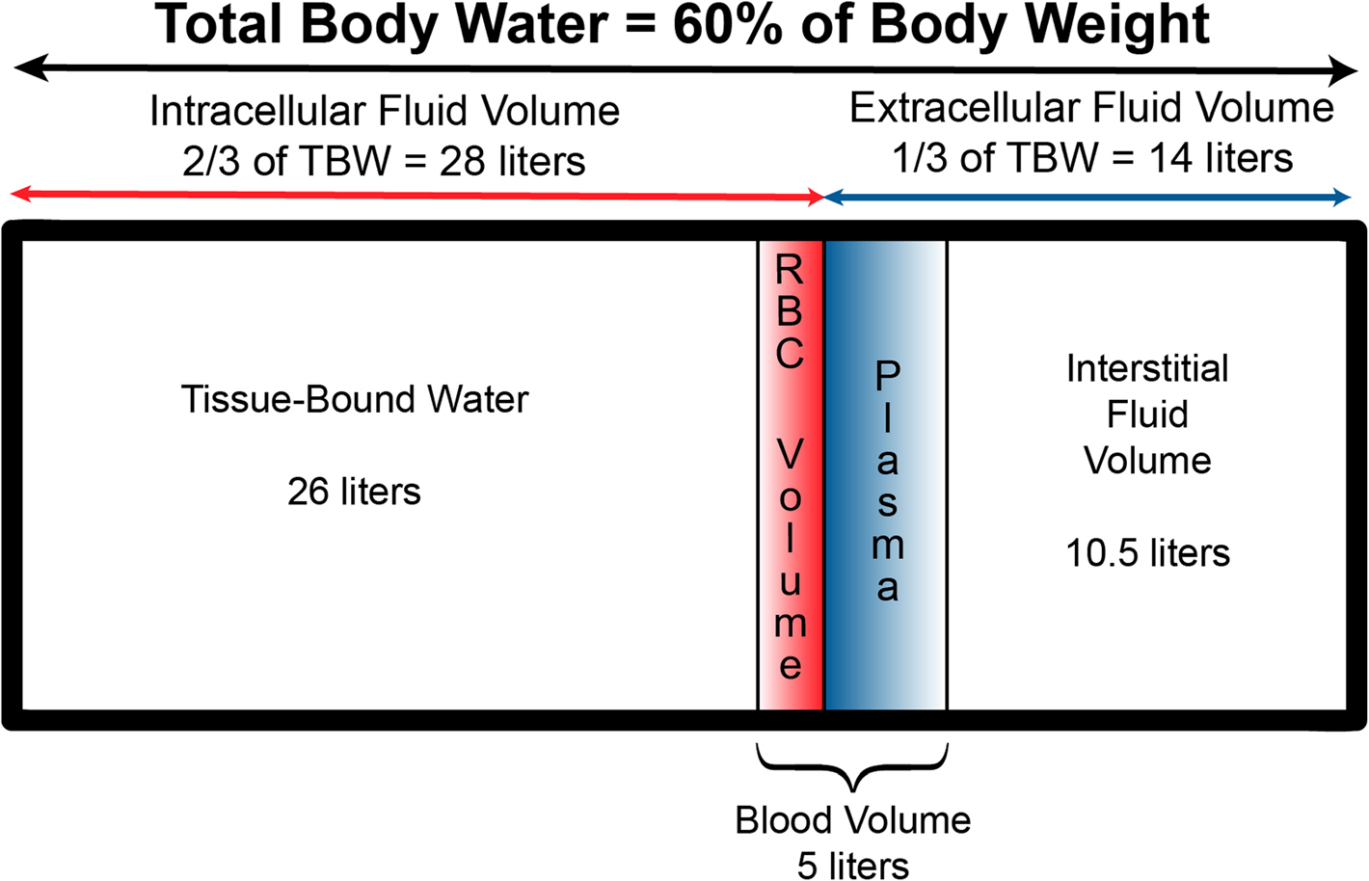
Fluid compartments in adult humans. Figure reused with the permission of the Perioperative Quality Initiative (POQI). For permission requests, contact info@poqi.org

Fluid optimization must consider each of the fluid compartments in the body: total body water divided into the intracellular and extracellular spaces, and more discretely the plasma volume and blood volume, the interstitial fluid volume, and the tissue-bound water volume (Fig. 2).

The left ventricle can only pump into the arterial circulation; the volume of blood that it receives from venous return (Funk et al., 2013). Because approximately two-thirds of the total blood volume is in the venous system, the roles of the venous system in general and venous capacitance specifically are important for fluid therapy and hemodynamic management. Through their capacitance function, veins and venules regulate both regional and central blood volume and therefore cardiac preload: changes in venous tone directly influence SV and CO via the Frank-Starling mechanism. It is important for anesthesia providers to understanding venous return, venous capacitance, and their role in determining CO.

**Fig. 3:**
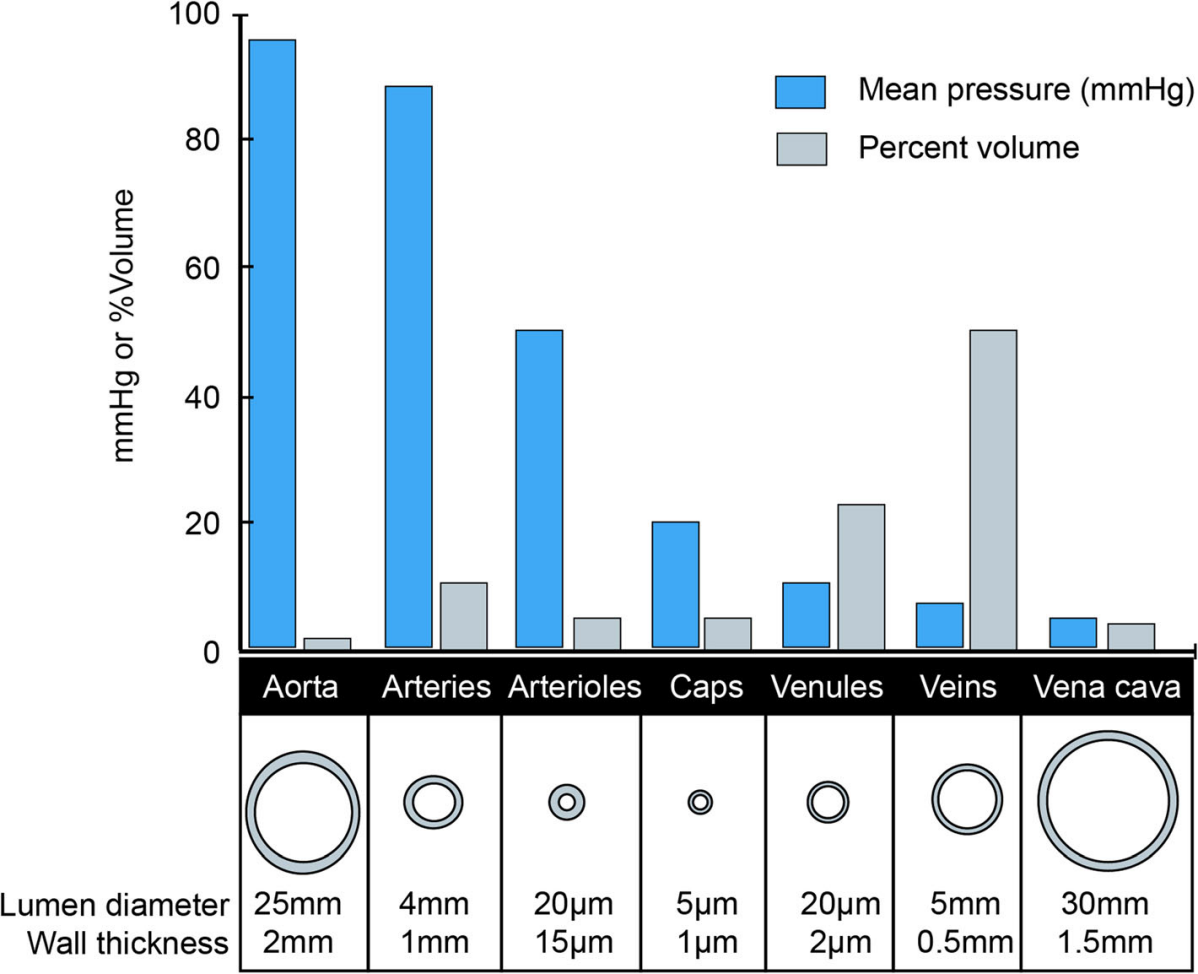
Pressure and volume in the venous system. Figure reused with the permission of the Perioperative Quality Initiative (POQI). For permission requests, contact info@poqi.org.

**Fig. 4:**
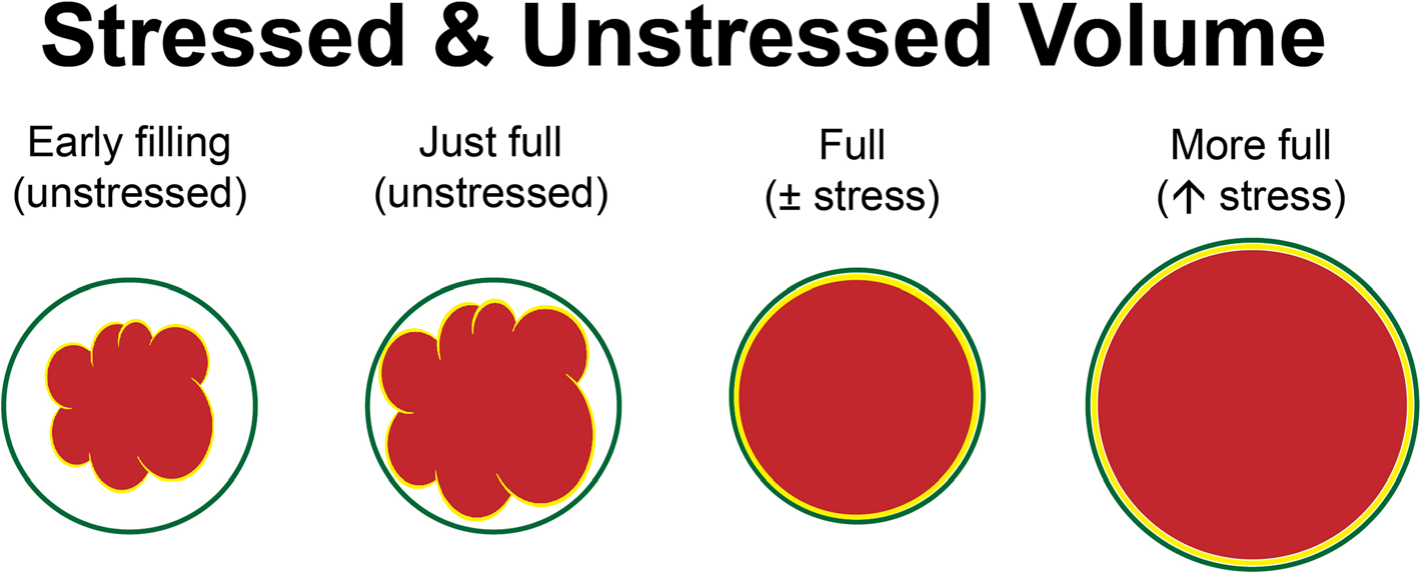
Depiction that differentiates stressed and unstressed volumes in the venous circulation. Figure reused with the permission of the Perioperative Quality Initiative (POQI). For permission requests, contact info@poqi.org

**Fig. 5:**
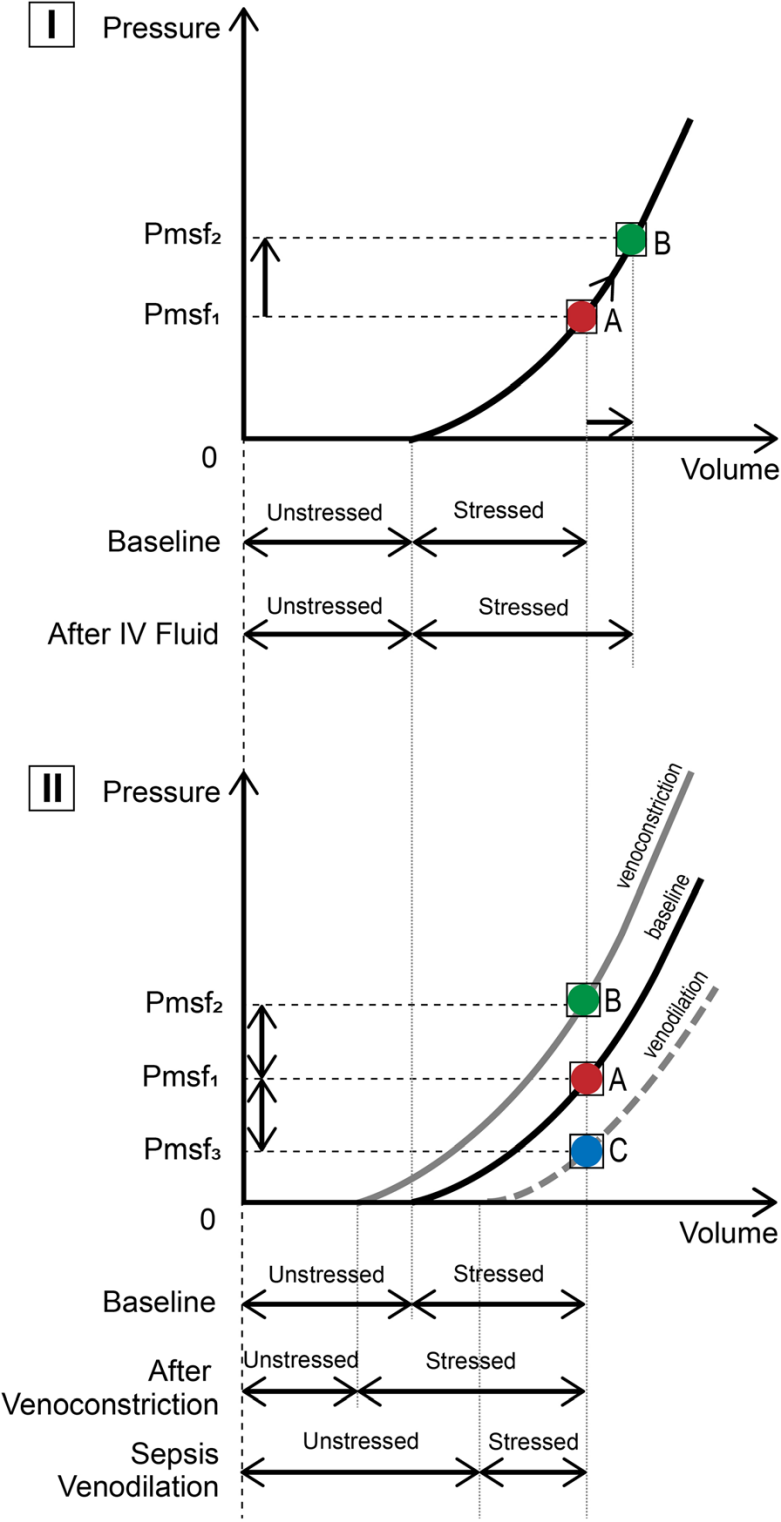
Effects of fluid and vasoactive agents on cardiovascular performance and the venous system. Figure reused with the permission of the Perioperative Quality Initiative (POQI). For permission requests, contact info@poqi.org. **I** Effect of volume loading on mean systemic filling pressure (Pmsf) and (un)stressed volume. Administration of a fluid bolus increases Pmsf (from Pmsf1 to Pmsf2, indicated respectively by position A (red dot) to B (green dot) on the pressure/volume curve). Unstressed volume remains constant while stressed volume increases. Total volume = unstressed + stressed increases, carrying a risk for fluid overload. See text for explanation. **II** Effect of venoconstriction and venodilation on mean systemic filling pressure (Pmsf) and (un)stressed volume. Venoconstriction increases Pmsf (from Pmsf1 to Pmsf2, indicated respectively by position A (red dot) to B (green dot) on the pressure/volume curve). Unstressed volume decreases while stressed volume increases. Total volume = unstressed + stressed remains constant, resulting in an auto-transfusion effect. Venodilation as seen in sepsis (vasoplegia) decreases Pmsf (from Pmsf1 to Pmsf3, indicated respectively by position A (red dot) to C (blue dot) on the pressure/volume curve). Unstressed volume increases while stressed volume decreases. Total volume = unstressed + stressed remains constant, resulting in an intravascular underfilling effect

With two-thirds of the blood volume within the venous system, changes in venous blood volume play a major role in determining venous return and CO (Fig. 3). The venous system can be divided into two theoretical compartments, the unstressed and the stressed volume (Gelman, 2008). The intravascular volume that fills the venous system to the point just before intravascular pressure starts to rise is called unstressed volume, whereas the volume that stretches the veins and causes intravascular pressure to rise is called the stressed volume (Figs. 4, 5). Another way to think of stressed volume is the (theoretically measurable) portion of blood that exerts distending pressure against the vein wall. Differentiating between these two volumes is important because stressed volume determines the MSFP, which is the pressure of venous return when cardiac activity is absent, representing the elastic recoil of the venous system. Through MSFP, the stressed blood volume is a major contributor of venous pressure and therefore venous return.

The unstressed volume normally accounts for about 75% of the venous blood volume, thus the venous system acts as a reservoir that can rapidly recruit blood from the unstressed blood volume to maintain venous return to the right heart (Gelman, 2008; Peters et al., 2001). Splanchnic and cutaneous veins are highly compliant and represent the largest blood volume reservoirs. Alterations in venous tone can change the relative proportions of the unstressed and stressed volumes. For example, alpha adrenergic receptor agonists may increase venous tone and thus increase the stressed volume (and simultaneously lower the unstressed volume), increasing venous return to the heart, raising SV and CO. (Kalmar et al., 2018; Hamzaoui et al., 2010)

#### What is fluid responsiveness? How do we define it, is there variation in proposed definitions, and is there variation from those in clinical practice?

Assessing fluid responsiveness is the safest and most effective method to guide fluid therapy. Conceptually, fluid responsiveness is defined as a state of recruitable SV in response to fluid administration (see Table 3).

Intravascular volume and other measures of volume status have limited utility and must always be considered in the context of fluid responsiveness. Intravascular volume is distinct from total body fluid volume and must be interpreted in the context of the individual patient. For example, patients with excess total body fluid volume may have normal or low intravascular volume and favorably respond to intravenous fluid administration. Common clinical terminology is shown in Table 3, and core concepts regarding volume status and fluid responsiveness include the following:

- All hypovolemic patients are fluid responsive but not all fluid responsive patients are hypovolemic.
- Euvolemic and hypervolemic patients may be fluid responsive (i.e., have preload recruitable SV). Therefore, fluid non-responsiveness does not indicate hypervolemia.
- The term fluid overload is confusing and is not the appropriate term to indicate intravascular hypervolemia (Vincent & Pinsky, 2018).

Clinically, fluid responsiveness is defined as an increase in SV in response to an increase in intravascular volume. Fluid responsiveness is one component of preload responsiveness, which indicates a state of recruitable SV and is defined as a state in which increases in end-diastolic volume (EDV) produce an increase in SV (Pinsky, 2015). At the bedside, there are varying definitions of fluid responsiveness based upon the setting and the monitoring available for the patient, with the most common definition being an increase of SV of 15% after the patient receives 500 mL of crystalloid administered over 10-15 min (Marik et al., 2009; Marik & Cavallazzi, 2013). Full characterization of fluid responsiveness requires consideration of the type, amount and timing of fluid, and the expected change in SV. It is noteworthy that even among hemodynamically unstable patients, only approximately half of patients are fluid responsive, which means that fluid loading in the approximately 50% of non-responsive patients will likely cause more harm than benefit.

**Fig. 6:**
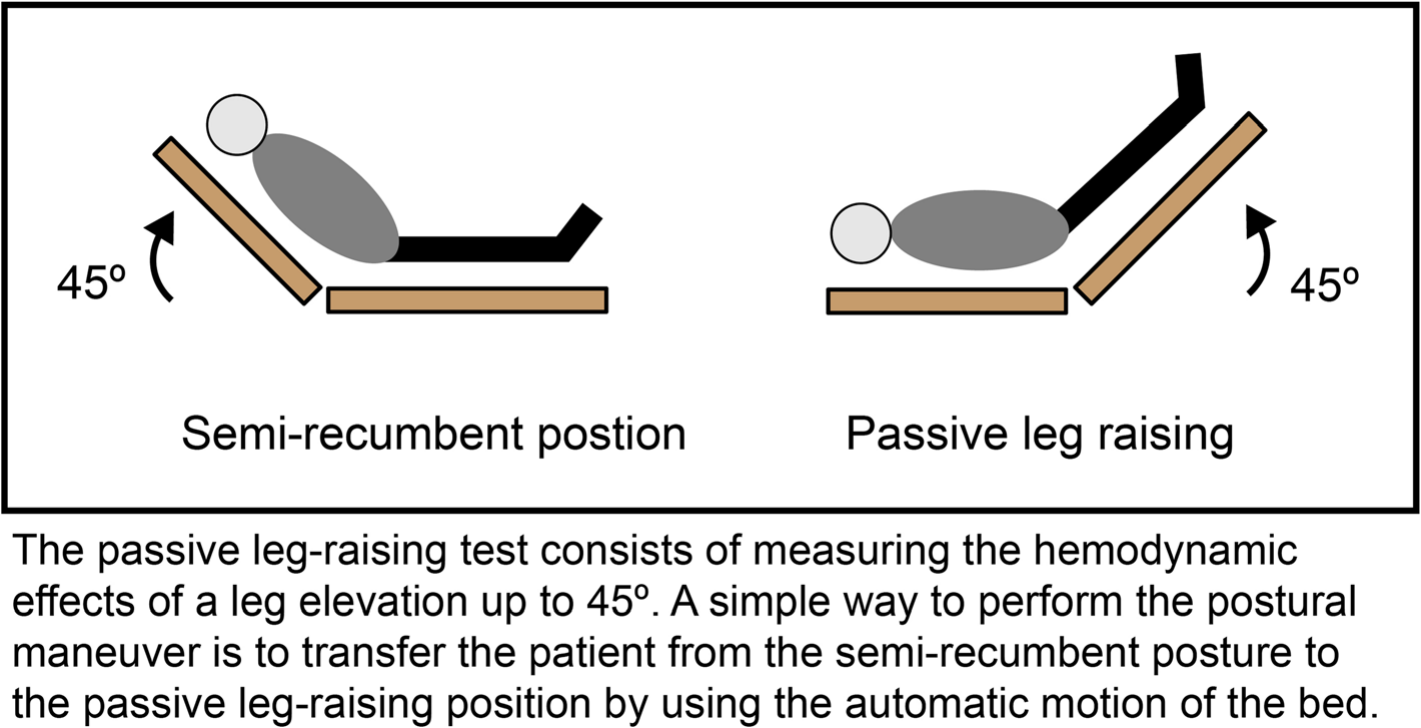
Stylized depiction of the passive leg raise (PLR) maneuver. Figure reused with the permission of the Perioperative Quality Initiative (POQI). For permission requests, contact info@poqi.org

The only method of directly measuring fluid responsiveness is continuous or rapidly repeatable measures of SV in response to a fluid challenge, a passive leg raise (PLR) maneuver, or controlled changes in intra-thoracic pressure. Passive leg raising is a postural maneuver raising the lower extremities up to 45 degrees from the recumbent position, which results in a transient increase in the venous return from the lower extremities in order to measure the hemodynamic effect and thus determine if a patient is responsive to fluid therapy (Fig. 6). Because PLR replicates a transient fluid bolus and predicts fluid responsiveness without administration of IV fluids, it mitigates the risk of excessive IV fluids that may be particularly deleterious in patients at greater risk for or poor tolerance of hypervolemia (e.g., heart failure, chronic kidney disease, chronic lung disease). Alternative methods for predicting fluid responsiveness include SVV, PPV, SPV, and (in certain mechanically ventilated patients) the end-expiratory occlusion test and respiratory systolic variation test (see Table 1).

A common approach to test fluid responsiveness is the administration of a 500 mL fluid challenge over < 15 min with a positive response defined by a ≥ 15% increase in SV, or a 250 mL fluid challenge over < 15 min with a positive response defined by a ≥ 10% increase in SV. However:

- Investigations into fluid responsiveness vary in fluid type, volume, infusion time, and consequent change in SV.
- Patients at risk for adverse effects from fluid administration, such as CHF and ESRD, may receive lower volume fluid challenges (e.g., 100 mL), although the accuracy of the test to predict fluid responsiveness is reduced (i.e., greater risk of false negative test).
- The volume of fluid challenge depends on the type of fluid given: crystalloid, colloid, or blood.

Recognizing that SV cannot be measured in all settings, MAP or HR may be used as crude surrogate indicators, recognizing they have limited predictive value. Although the existing literature has examined the effect of a fluid challenge on SV, a fluid challenge or PLR could be useful in detecting whether inadequate preload is contributing to hypotension. If the fluid challenge/PLR does not correct hypotension, additional monitoring may be appropriate and further management should focus on vascular tone and chronotropy/inotropy (McEvoy et al., 2019).

#### Under what situations can fluid responsiveness be used clinically to decide when to give, and when to stop giving fluid?

In the perioperative period, when fluid losses may be substantial, fluid responsiveness is generally an indication for fluid administration but should be interpreted in the clinical context of the patient. In patients who are predicted to be non-responsive to fluid administration, fluids should not be given unless other clinical indicators suggest net benefit (e.g., need for water). The same assessments apply to the decision to stop giving intravenous fluids, which may be based upon the parameters judging fluid responsiveness and the clinical context of the patient for a global assessment or benefit and risk.

A key clinical question to ask when assessing a patient’s fluid responsiveness is whether increasing the SV and CO is beneficial. For example, a patient might have an adequate or high SV index and cardiac index and still show evidence of fluid responsiveness. Giving fluid might not be indicated since achieving a still higher SV index and cardiac index might not yield important clinical benefits.

All tests for fluid responsiveness have limitations (Table 1). For example, SVV testing requires a regular cardiac rhythm, the lack of patient inspiratory efforts, and consistent changes in intrathoracic pressure produced by mechanical ventilation with tidal volumes of at least 8 mL/kg predicted body weight. PLR testing requires measurement of SV or CO and may produce false negative results in the setting of intra-abdominal hypertension. While ultrasonographic evaluation of vena cava diameter, distensibility, and collapsibility are increasingly used in emergency medicine and critical care, its use has numerous confounders (right heart dysfunction, obstructive cardiac physiology, transpulmonary and intraabdominal pressure, mode of ventilatory support) and is not currently supported by evidence in the perioperative setting (Millington, 2019; Via et al., 2016). Finally, it is important to note that fluid bolus therapy rapidly impacts the macrocirculation but does not necessarily alter the microcirculation or cellular function, especially during the short time frame used to assess fluid responsiveness. Further, the effects of crystalloid boluses on SV and CO are often short-lived, as the crystalloid fluid redistributes into the extravascular extracellular space (Nunes et al., 2014; Aya et al., 2016).

Despite these limitations the assessment of fluid responsiveness probably leads to improved patient outcomes among patients who undergo high-risk and complex surgery (Bednarczyk et al., 2017).

#### What is the research agenda?

Our increasing understanding of the physiological and clinical consequences of intravenous fluid therapy has led to new and important questions that must be answered in order to further refine our clinical use of intravenous fluids. Future research should focus on the following key areas:

- As the fundamental therapeutic goal of intravenous fluid administration into the macrocirculation is to optimize microcirculatory and cellular function, we need better tools to assess both of those critical features at the bedside.
- We need to identify or create methods with everyday clinical utility to measure intravascular volume, and for monitoring the benefits/harm of fluid therapy.
- We need to better define exactly how to perform a fluid challenge and a fluid responsisve patient.
- Emerging evidence suggests that fluid management should always be individualized based on each patients unique hemodynamics and cardiac funcion, underlying disease process and co-morbidities. We must recognize the dynamic nature of patient trajectories throughout the perioperative period to better define optimal fluid administration and removal strategies.

Further research in these key areas will lay the foundation for moving from group-targeted fluid therapy to truly individualized fluid therapy.

## Conclusions

In the POQI-5 consensus conference, we discussed the clinical and physiological evidence of fluid responsiveness and venous capacitance as relevant factors in fluid management and developed consensus statements with clinical implications for a broad group of clinicians involved in intravenous fluid therapy. Two key concepts emerged: (1) The ultimate goal of fluid therapy and hemodynamic management is to provide the conditions that enable normal cellular metabolic function in order to produce optimal patient outcomes, and (2) fluid and hemodynamic management is dependent on the relationship between pressure, volume, and flow in a dynamic system which is distensible and has variable elastance and capacitance.

## Data Availability

Not applicable

## Abbreviations

CVP: Central venous pressure
CO: Cardiac output
EDV: End-diastolic volume
GDT: Goal-directed therapy
HR: Heart rate
IV: Intravenous
MAP: Mean arterial pressure
MSFP: Mean systemic filling pressure
PLR: Passive leg raise
POQI: Perioperative Quality Initiative
PPV: Pulse pressure variation
SPV: Systolic pressure variation
ScvO2: Mixed central venous oxygen saturation
SvO2: Mixed venous oxygen saturation
SV: Stroke volume
SVV: Stroke volume variation
